# Vaccine hesitancy and the willingness to recommend the COVID-19 vaccine to children in a rural country on the United States-Mexico border

**DOI:** 10.3389/fpubh.2023.1127745

**Published:** 2023-05-03

**Authors:** Raghu D. Darisi, Audrey J. Buckland, Mario Morales, Maia Ingram, Emily Harris, Jeffrey R. Holzberg

**Affiliations:** ^1^Chiricahua Community Health Centers, Inc., Douglas, AZ, United States; ^2^Department of International Health, Johns Hopkins Bloomberg School of Public Health, Baltimore, MD, United States; ^3^Health Behavior Health Promotion, University of Arizona, Tucson, AZ, United States; ^4^Arizona Prevention Research Center, College of Public Health, University of Arizona, Tucson, AZ, United States

**Keywords:** COVID-19, vaccine willingness, vaccine hesitancy, United States-Mexico border, children, pediatrics

## Abstract

**Introduction:**

As of October 26, 2022, only 9% of children in the United States aged 6 months to 4 years have received at least one dose of COVID-19 vaccine despite FDA approval since June 17, 2022. Rates are better yet still low for children aged 5 to 11 years as nearly 30% were fully vaccinated as of August 23, 2022. Vaccine hesitancy among adults is one of the major factors affecting low vaccine uptake rates in children against COVID-19, yet most studies examining vaccine hesitancy have targeted school-age and adolescent children.

**Methods:**

With the aim of assessing the willingness to recommend the COVID-19 vaccination to children under 5 years compared to children 5 to 12 years of age, a county-wide survey was conducted between January 11 and March 7, 2022, among adults on the United States-Mexico border.

**Results:**

Among the 765 responses, 72.5% were female and 42.3% were Latinx. The most significant factor associated with likelihood to recommend the COVID-19 vaccine to children less than 5 years and 5–12 years of age was adult vaccination status. Ordinal logistic regression also indicated that ethnicity, primary language, being a parent, previous COVID-19 infection, and concern about getting COVID-19 in the future were significantly associated with likelihood of COVID-19 vaccine recommendation to children < 5 years and 5–12 years old.

**Discussion:**

This study found high consistency among respondents in their willingness to vaccinate children aged < 5 years compared with children aged 5–12 years. Our findings support public health strategies that target adult vaccinations as an avenue to improve childhood vaccinations for young children

## Background

In the United States, approximately 14.3 million children have tested positive with COVID-19 infection as of August 2022, and nearly 6.4 million tested positive in 2022 alone (1). Of the total COVID-19 cases reported in the United States up to August 2022, 6.6% were children aged 5 to 11 years, and 3.5% were 0 to 4 years of age (2). COVID-19 infection in children typically presents with fever and cough as the main symptoms, although gastrointestinal symptoms such as vomiting and diarrhea are more common in children than adults (3–5). Despite an estimated 4% of pediatric cases requiring intensive care unit admission and higher critical illness in infants under 6 months of age, the risk of hospitalization and life-threatening events are low in children infected with COVID-19 (6–8). Co-infection within the family is the primary route of transmission for pediatric COVID-19, highlighting the risk of children transmitting the virus to adults who have higher rates of morbidity and mortality with infection (3, 4). The risk of COVID-19 transmission from children is heightened by a longer incubation period, longer respiratory and fecal shedding, and higher RNA load compared to adults (9, 10).

The COVID-19 vaccine for children aged 5 to 11 years was authorized by the U.S Food & Drug Administration (FDA) for emergency use on October 29, 2021 (11). Then, on June 17, 2022, the FDA issued Emergency Use Authorization for the Moderna and Pfizer-BioNTech COVID-19 vaccines for children as young as 6 months of age (12). Despite robust vaccination awareness efforts, only 29.6% of children aged 5 to 11 years in the U.S. were fully vaccinated as of August 23, 2022 (13). As of October 26, 2022, only 9% of children aged 6 months to 4 years have received at least one dose of COVID-19 vaccine. Systematic reviews and meta-analyzes show an overall parental willingness to vaccinate their children against COVID-19 between 59 and 72%, and parental refusal of vaccination between 8 and 22% (14–16). Hesitancy to vaccinate children against COVID-19 is consistently higher among parents with poor vaccine knowledge and parents who are not vaccinated themselves, with differences in race and ethnicity not consistently found in studies (15–20). The most commonly reported concerns among vaccine-hesitant parents to vaccinate their children include vaccine side effects, that the vaccine was developed too fast, and that COVID-19 infection is less of a health threat for children compared to adults (15, 16, 18, 21–23).

Theoretical frameworks for health behaviors and decision-making, such as the Health Belief Model (HBM) (24–26) and Theory of Planned Behavior (TPB) (27, 28), have been used to explain and predict vaccine hesitancy, with specific application to COVID-19 vaccine intention and hesitancy (29–34). Applying the extended HBM to vaccine intention and hesitancy recognizes an individual’s perceived susceptibility to and severity of COVID-19 infection, perceived benefits and risks of getting a COVID-19 vaccine, and self-efficacy to get a COVID-19 vaccine to inform their vaccination behavior (35–37). The TPB recognizes that intention is the immediate antecedent to getting the vaccine and proposes that intent to get the COVID-19 vaccine can be predicted from and influenced by attitude, norms and perceived behavioral control (27, 28). While the HBM and TPB have been primarily used to examine the hesitancy and willingness of adults to get vaccinated, these behavioral models also provide a sound theoretical grounding for examining adults’ willingness to recommend the COVID-19 vaccine for children.

Most studies examining COVID-19 vaccine hesitancy among parents have focused on school-age and adolescent children (22, 38–42). At the time of this publication, only a few studies have examined vaccine hesitancy among parents of children under 5 years in the United States, and these studies were limited in participant racial and ethnic diversity (18, 43–46). The COVID-19 pandemic has exacerbated existing racial and ethnic health disparities; more specifically, the Latinx population in the US has experienced higher rates of COVID-19 disease incidence, hospitalization, and mortality compared to non-Latinx, white Americans (47–50). These disparities in morbidity and mortality are likely driven by the inequitable access to culturally preferred healthcare services (51), limited Spanish proficiency among healthcare providers and restricted access to COVID-19 information due to English-only communication materials (51–53), inequitable access to COVID-19 testing (54), challenges to comply with social distancing protocols due to employment constraints (55, 56) and, for some, fears of jeopardizing immigration status (57). No published studies have focused on assessing COVID-19 vaccine hesitancy among Latinx communities or compared vaccine willingness for children under 5 years with children aged 5 to 12 years. Even further, no studies have compared parents with non-parents in their willingness to vaccinate children within these age groups.

Among adults in a rural county on the United States-Mexico border, this study aims to: (1) assess the prevalence of reported willingness to recommend the COVID-19 vaccination for children under 5 years of age and children 5 to 12 years of age, (2) identify the factors associated with COVID-19 vaccine willingness for children of these two age groups, and (3) characterize concerns for vaccinating children of these two age groups.

## Methods

### Study design

Cochise County is a rural county located in southeastern Arizona that shares its southern border with Mexico. 54% of the county population is White and Non-Hispanic, while 36% is Latinx and 5% African American (58). The second week of January 2022, when this study began, Cochise County had more diagnosed cases of COVID-19 than any other week since the pandemic started (59). Chiricahua Community Health Centers, Inc. (CCHCI) is a diverse, not-for-profit, Federally Qualified Community Health Center that provides both primary and enabling services for adults and pediatrics with multiple locations across the county. CCHCI is also one of the largest providers of the COVID-19 vaccine in Cochise County. A cross-sectional study was conducted utilizing a paper and online survey distributed by CCHCI throughout the county to understand barriers to vaccine acceptance as COVID-19 infection rates continued to rise.

The survey was based on the Rapid Community Assessment Guide by the Centers for Disease Control and Prevention (CDC) (60). The research team determined the final set of questions based on CCHCI’s research interests, previously published vaccine hesitancy studies, and to limit the response burden for participants. The survey comprised of three sections: sociodemographics, health-related questions, and COVID-19-specific questions. The sociodemographics section included age group, gender, race/ethnicity, primary spoken language, number and age of children, setting of residence (urban, semiurban, and rural), zip code, education, and current industry of work. The health-related section included questions on health insurance, primary care provider, and physical or emotional conditions preventing them from activities of daily life. The COVID-19-specific section included questions on COVID-19 vaccination status (i.e., fully vaccinated, partially vaccinated, and unvaccinated), reasons for not getting vaccinated (among the unvaccinated and partially vaccinated), booster dose status, previous COVID-19 infection for self or family member, concern of getting COVID-19 in the future, and their top trusted sources of information on the COVID-19 vaccine.

#### Data collection

We reached out to local stakeholders, community organizations, community groups, universities, and other county contacts for distribution of the survey through their networks in Cochise County. Flyers with a QR code in both English and Spanish were displayed at front desks, notice boards and information walls of public places in Cochise County. Surveys were also distributed to the patient population at CCHCI, who agreed to receive surveys from our organization. The survey was opened on January 11, 2022, and ended on March 7, 2022. Online survey distribution used the Survey Monkey platform and paper surveys were entered manually. The survey was offered in both English and Spanish. Inclusion criteria includes all adults currently living in Cochise County who are 18 years of age or older. Consent was obtained on the first page of the and additional questions would only be answered if the participant met the inclusion criteria of being at least 18 years old. Ethics approval from an Institutional Review Board was not required for this study as it was part of a quality improvement project at CCHCI.

#### Measures

The primary outcome of interest was measured by the item: “*If a COVID vaccine were available for children younger than 5 years of age (or 5–12 years of age), how likely are you to recommend the vaccine?*” This ordinary variable was self-reported as “not at all likely,” “somewhat likely,” and “extremely likely.” Independent variables included COVID-19 related characteristics (i.e., being concerned about getting COVID-19, previously having COVID-19, and having family members with COVID-19). Control variables included sociodemographic characteristics (i.e., gender, age, ethnicity, primary language, number of children, and location).

COVID-19 related characteristics were measured with three items: “*How concerned are you about getting COVID infection in the future?*,” “*To your knowledge, do you have, or have you been infected with COVID?*” and “*Has anyone in your family had their health be significantly affected by COVID infection?*.” The four categories of the first variable were collapsed as follow: not at all concerned/a little concerned, and moderately concerned/very concerned. The second and third variables comprised two categories: no and yes.

Sociodemographic characteristics were measured with six items: “*Gender*,” “*Age*,” “*Ethnicity*,” “*Primary spoken language*,” “*Number of children you have*,” and “*Location.*” The first variable included three categories: man, woman, and I would rather not say; it was grouped in two categories: men and women (the third category included only three observations that were excluded from the analysis). The second variable comprised six categories (i.e., 18–24 years, 25–34 years, 35–44 years, 45–54 years, 55–64 years, and 65 and above) that were collapsed as follows: 18–44, 45–64, and >64. The third variable included three categories: Hispanic or Latino, not Hispanic or Latino, and Other; it was grouped in two categories: Latinx and Non Latinx (the latter collapsed not Hispanic or Latino, and other). The fourth variable comprised three categories (i.e., English, Spanish, and other); the three observations of the latter were deleted because the focus of the analysis was English and Spanish differences. The fifth variable included four categories: number of children less than 5, number of children between 5 and 12, number of children between 13 and 17, and number of children above 17; it was grouped in two categories (presence or absence of children); the last variable comprised three categories (i.e., urban, suburban, and rural).

#### Statistical analysis

The analysis used data from the Chiricahua COVID-19 vaccine hesitancy survey. An exploratory data analysis was conducted to compare the demographic, health-related, and COVID-19-specific characteristics of participants by self-reported likelihood of recommending the COVID-19 vaccine to children <5 years of age and children 5–12 years of age. Kruskal–Wallis tests by rank were used for the comparisons of all categorical variables. Next, two ordinal outcomes were parameterized: (1) self-reported likelihood of recommending the COVID-19 vaccine for children <5 years of age comparing extremely likely vs. somewhat or not likely and (2) self-reported likelihood of recommending the COVID-19 vaccine for children between 5 and 12 years of age comparing extremely likely vs. somewhat or not likely.

Multivariable ordinal logistic regression was used to analyze the association between respondent demographics and health-related information and the two ordinal outcomes of interest to identify respondent characteristics associated with the recommendation of the COVID-19 vaccine for children <5 years of age, and children between 5 and 12 years of age. Demographic, health-related, and COVID-19-specific characteristics were considered for the final model if there were sufficient observed cases in the categories (*n* > 5), which eliminated vaccine status from consideration. However, the most endorsed concerns reported by participants for vaccinating children in both age groups were not considered for inclusion in the model because respondents could select multiple responses, and thus operationalization of the variable is less interpretable.

The log likelihood test statistic was used to assess model fit and to compare nested models. Potential confounders were considered for inclusion in the multivariable model. Multicollinearity was assessed and ruled out upon obtaining appropriate values for the variance inflation factors (VIF). The models showed VIF values lower than 1.6, which indicated a low to moderate correlation between the predictors. Ethnicity and primary language were considered independent variables in the final model given the low VIF and because not all Latinx individuals living in Cochise County are Spanish speakers. Predictive mean matching was performed to impute missing data (i.e., it randomly filled values from observed donor values using the Mice package in R 4.1); five imputations were combined in an original observed data.

## Results

A total number of 1,014 survey responses were recorded. Records with completely missing data (*n* = 249) were removed from the analysis. Records with partially missing or complete data were included in the analytic sample (*n* = 765).

Table 1 presents the overall demographic characteristics of survey respondents and the reported likelihood of recommending the COVID-19 vaccine to children. The majority of our sample were female (72.9%), English-speaking as the primary language (82.7%), and had children (76.1%). The most common age groups of survey respondents were 45–64 years old (40.9%), followed by 18–44 years (36.7%), and ≥65 years (22.4%). Latinx and non-Latinx represented 42.3 and 57.7% of our sample, respectively. The majority of respondents lived in rural areas (66.7%), followed by suburban (17.7%), and urban (15.6%) areas.

**Table 1:** Participant demographic characteristics by vaccine recommendation (not likely vs. somewhat likely vs. extremely likely) for children <5 years (*n* = 758) and children 5–12 years in 2022 (*n* = 759).

Variable	For children <5 years				For children 5–12 years			
	Not likely	Somewhat likely	Extremely likely	*p*-value^a^	Not likely	Somewhat likely	Extremely likely	*p*-value^a^
	(*N* = 296)	(*N* = 192)	(*N* = 270)		(*N* = 263)	(*N* = 128)	(*N* = 368)	
Gender				0.364				0.185
Male	87 (29.6%)	52 (27.1%)	65 (24.3%)		81 (30.9%)	34 (26.8%)	89 (24.3%)	
Female	207 (70.4%)	140 (72.9%)	203 (75.7%)		181 (69.1%)	93 (73.2%)	277 (75.7%)	
Age group (years)				0.244				0.551
18–44	117 (39.5%)	61 (31.8%)	99 (36.9%)		100 (38.0%)	44 (34.4%)	132 (36.0%)	
45–64	120 (40.5%)	87 (45.3%)	101 (37.7%)		109 (41.4%)	51 (39.8%)	151 (41.1%)	
≥65	59 (19.9%)	44 (22.9%)	68 (25.4%)		54 (20.5%)	33 (25.8%)	84 (22.9%)	
Ethnicity				<0.001				<0.001
Non Latinx	201 (67.9%)	106 (55.2%)	132 (49.1%)		186 (70.7%)	67 (52.3%)	186 (50.7%)	
Latinx	95 (32.1%)	86 (44.8%)	137 (50.9%)		77 (29.3%)	61 (47.7%)	181 (49.3%)	
Primary language				<0.001				<0.001
English	273 (92.2%)	152 (79.2%)	202 (75.1%)		244 (92.8%)	95 (74.8%)	286 (77.9%)	
Spanish	23 (7.8%)	40 (20.8%)	67 (24.9%)		19 (7.2%)	32 (25.2%)	81 (22.1%)	
Location				0.968				0.446
Rural	194 (65.8%)	129 (67.2%)	180 (66.9%)		171 (65.3%)	80 (62.5%)	254 (69.0%)	
Urban	49 (16.6%)	28 (14.6%)	41 (15.2%)		44 (16.8%)	24 (18.8%)	50 (13.6%)	
Suburban	52 (17.6%)	35 (18.2%)	48 (17.8%)		47 (17.9%)	24 (18.8%)	64 (17.4%)	
Parent				0.003				0.005
No	50 (17.3%)	50 (26.7%)	75 (29.3%)		47 (18.4%)	25 (20.3%)	103 (29.2%)	
Yes	239 (82.7%)	137 (73.3%)	181 (70.7%)		209 (81.6%)	98 (79.7%)	250 (70.8%)	
COVID-19 vaccination status				<0.001				<0.001
Complete^b^	139 (47.0%)	177 (92.7%)	265 (98.5%)		111 (42.2%)	110 (86.6%)	361 (98.4%)	
Partial^c^	19 (6.4%)	10 (5.2%)	3 (1.1%)		16 (6.1%)	12 (9.5%)	4 (1.1%)	
Unvaccinated^d^	138 (46.6%)	4 (2.1%)	1 (0.4%)		136 (51.7%)	5 (3.9%)	2 (0.5%)	
Previous COVID-19 infection				<0.001				<0.001
No	147 (49.7%)	126 (65.6%)	187 (69.3%)		133 (50.6%)	79 (61.7%)	249 (67.7%)	
Yes	149 (50.3%)	66 (34.4%)	83 (30.7%)		130 (49.4%)	49 (38.3%)	119 (32.3%)	
Family member affected by COVID-19				0.852				0.839
No	201 (68.4%)	135 (70.7%)	184 (68.7%)		177 (67.8%)	88 (69.3%)	257 (70.0%)	
Yes	93 (31.6%)	56 (29.3%)	84 (31.3%)		84 (32.2%)	39 (30.7%)	110 (30.0%)	
Concerned about getting COVID-19				<0.001				<0.001
Not at all/a little	220 (74.6%)	80 (41.7%)	60 (22.2%)		203 (77.5%)	58 (45.3%)	99 (26.9%)	
Moderately/Very	75 (25.4%)	112 (58.3%)	210 (77.8%)		59 (22.5%)	70 (54.7%)	269 (73.1%)	

a *p*-values were calculated from Chi-squared tests.

b Not including booster.

c Received at least two doses of Pfizer/Moderna or one dose of Johnson & Johnson.

d Received one dose of Pfizer/Moderna.

Most respondents were partially or completely vaccinated against COVID-19 (81.3%). Only 39.2% of respondents were previously infected with COVID-19, and 30.8% had a family member with a significant COVID-19 infection in the past. Of 765 participants in the survey, only 78% of them answered the question on their concern about getting COVID-19 in the future. However, 39.2% of them were moderately or very concerned about getting COVID-19, with 60.8% expressing little or no concern.

### Likelihood of vaccine recommendation to children

As seen in Table 1, 39.1% of survey respondents were not likely (NL), 25.3% were somewhat likely (SL), and 35.6% were extremely likely (EL) to recommend the COVID-19 vaccine to children under 5 years old. There were no statistically significant differences in the distribution of respondent gender, age group, or location between NL, SL, and EL COVID-19 vaccine recommending groups.

Ethnicity was found to be significantly associated with respondents’ reported likelihood of recommending the COVID-19 vaccine for children under 5 years of age (*p* < 0.001) and for children 5–12 years of age (*p* < 0.001). Ethnicity was more evenly distributed among participants who were EL to recommend the COVID-19 vaccine to children under 5 years of age (49.1% non-Latinx vs. 50.9% Latinx), but a higher proportion of those who were NL to recommend the vaccine were non-Latinx (67.9%) compared to Latinx (32.1%). Similarly, half of the respondents who were EL to recommend the COVID-19 vaccine to children 5–12 years of age were non-Latinx, but 70.7% of those NL to recommend the vaccine were non-Latinx.

Adult vaccination status was significantly associated (*p* < 0.001) with respondents’ reported likelihood of recommending the COVID-19 vaccine for children under 5 years and 5–12 years, as nearly all (98.5% for children under 5 and 98.4% for children 5–12 years) of respondents who were EL to recommend the COVID-19 vaccine were completely vaccinated. Furthermore, most participants who were SL to recommend the vaccination for children under 5 years and 5–12 years were also completely vaccinated (92.7 and 86.6%, respectively). Among those who were NL to recommend the COVID-19 vaccine for children under 5 years, 47.0% were completely vaccinated, 6.4% were partially vaccinated, and 46.6% were unvaccinated. Similarly, among those who were NL to recommend the COVID-19 vaccine for children 5–12 years of age, 42.2% were completely vaccinated, 6.1% were partially vaccinated, and 51.7% were unvaccinated.

No significant differences were observed in the reported likelihood of recommending the COVID-19 vaccine for children among respondents who had a family member who was seriously infected with COVID-19 versus those who did not. However, significant differences in the reported likelihood of recommending the COVID-19 vaccine to children were observed between adults who had been previously infected with COVID-19 and those who had not been previously infected. Of those who were EL and SL to recommend the COVID-19 vaccine for children under 5 years, a lower proportion (EL 30.7% and SL 34.4%) of them had been infected with COVID-19. However, the proportion of respondents with previous COVID-19 among NL is nearly equal (50.3% vs. 49.7%) compared to respondents without previous COVID-19 infection. This is also similar with the groups who were EL and SL to recommend the COVID-19 vaccine for children 5–12 years of age, where only 32.3% (EL) and 38.3% (SL) of them had been infected with COVID-19 in the past. Among NL, the proportion of respondents with previous COVID-19 is nearly equal (50.6% vs. 49.4%) to respondents without previous COVID-19 infection.

There were also differences in the reported likelihood of recommending the COVID-19 vaccine to children based on concern about getting infected with COVID-19, with respondents who were not at all or a little concerned being significantly less likely to recommend the COVID-19 vaccine to children <5 years of age (NL 74.6%, SL 41.7%, EL 22.2%; value of *p* <0.001) and to children 5–12 years of age (NL 77.5%, SL 45.3%, EL 26.9%; value of *p* <0.001). A significant association was also found in the reported likelihood of recommending the COVID-19 vaccine to children under 5 years and 5–12 years comparing parents to non-parents.

### Multivariable ordinal logistic regression

As described above, we observed a strong association between respondent vaccination status and their reported likelihood of recommending the COVID-19 vaccine for children. However, vaccination status was not included in the logistic regression models because it does not contain enough cases in all categories to ensure precise effect estimates. Table 2 presents the adjusted OR for the associations between participant characteristics and being EL to recommend the COVID-19 vaccine vs. SL or NL to recommend the vaccine to children.

**Table 2:** Multivariable ordinal logistic regression of characteristics associated with participants who report being extremely likely vs. somewhat or not likely to recommend the COVID-19 vaccine to children.

Characteristics	Children <5 years (*n* = 758)		Children 5–12 years (*n* = 759)	
	Odds ratio (OR)	95% CI	Odds ratio (OR)	95% CI
Gender				
Male (ref)	–	–	–	–
Female	1.08	0.78–1.50	1.14	0.82–1.61
Age group (years)				
18–44 years (ref)	–	–	–	–
45–64 years	0.92	0.66–1.29	0.98	0.69–1.38
≥65 years	1.20	0.80–1.82	1.1	0.66–1.55
Ethnicity				
Non-Latinx (ref)	–	–	–	–
Latinx	1.50*	1.04–2.15	1.64*	1.12–2.39
Primary language				
English (ref)	–	–	–	–
Spanish	2.20***	1.41–3.45	1.77*	1.10–2.84
Location				
Rural (ref)	–	–	–	–
Urban	1.02	0.67–1.54	0.87	0.57–1.33
Suburban	1.27	0.87–1.87	1.18	0.79–1.75
Presence of children				
No (ref)	–	–	–	–
Yes	0.62*	0.44–0.89	0.59*	0.41–0.86
Prior COVID-19 infection				
No (ref)	–	–	–	–
Yes	0.51***	0.37–0.69	0.57***	0.41–0.78
Family member affected by COVID-19				
No (ref)	–	–	–	–
Yes	0.94	0.68–1.30	0.74	0.52–1.04
Concerned about getting COVID-19				
Little/Not concerned (ref)	–	–	–	–
Moderately/Very concerned	5.70***	4.21–7.72	5.92***	4.34–8.08

*p < 0.05, **p < 0.01, ***p < 0.001.

Holding all other variables constant, respondents who were moderately/very concerned about getting COVID-19 had 470% higher odds of being EL (vs. SL or NL) to recommend the vaccine to children <5 years of age (aOR = 5.70, 95% CI 4.21–7.72) and 492% higher odds of being EL (vs. SL or NL) to recommend the vaccine to children 5–12 years of age (aOR = 5.92, 95% CI 4.34–8.08) compared with respondents who are little/not at all concerned about getting COVID-19 in the future. In contrast, holding all other variables constant, compared to respondents not previously infected with COVID-19, respondents who had been previously infected with COVID-19 had 49% lower odds of being EL (vs. SL or NL) to recommend the vaccination for children <5 years (aOR = 0.51, 95% CI 0.37–0.69) and 44% lower odds of being EL (vs. SL or NL) to recommend it for children 5–12 years old (aOR = 0.57, 95% CI 0.41–0.78).

After adjusting for all other variables, compared to non-Latinx respondents, Latinx respondents had 50 and 64% higher odds of being EL (vs. SL or NL) to recommend COVID-19 vaccination to children <5 years of age and 5–12 years of age, respectively (aOR = 1.50, 95% CI 1.04–2.15; aOR = 1.64, 95% CI 1.12–2.39, respectively). Similarly, respondents with Spanish as their primary language had 120 and 77% higher odds of being EL (vs. SL or NL) to recommend COVID-19 vaccination to children <5 years of age and 5–12 years of age, respectively, compared to respondents with English as their primary language (aOR = 2.20, 95% CI 1.41–3.45; aOR = 1.77, 95% CI 1.10–2.84, respectively). Holding all other variables constant, parents had 38% lower odds of being EL (vs. SL or NL) to recommend the COVID-19 vaccination to children <5 years old (aOR = 0.62, 95% CI 0.44–0.89) and 41% lower odds of being EL (vs. SL or NL) to recommend the COVID-19 vaccination to children 5–12 years old (aOR = 0.59, 95% CI 0.41–0.78), compared to non-parents.

After controlling for all other predictors, no significant differences were found in the odds of being EL vs. SL or NL to recommend the COVID-19 vaccine to children according to gender, age group, location, or having a significant COVID-19 infection in the family.

### Comparison of COVID-19 vaccine recommendation to children under 5 years old and children 5–12 years old

One in three respondents (34.1%) were extremely likely to recommend the COVID-19 vaccine to children <5 years and children 5–12 years of age, as well as not at all likely to recommend the vaccine to both age groups; one in nine (11.0%) were somewhat likely to recommend the vaccine to both age groups (Table 3). Thus, 78.7% of respondents were consistent in their willingness to vaccinate children of both age groups, while 21.3% expressed a different likelihood of vaccine recommendation based on the child’s age. Respondents who were not fully vaccinated were the most unchanged in their willingness to vaccinate children regardless of age group; 96.0% expressed the same willingness to recommend to both groups. Respondents who were fully vaccinated, on the other hand, were the group who changed the most in their willingness to vaccinate children against COVID-19, as only 73.4% expressed the same willingness to recommend it regardless of age group.

**Table 3:** Comparison of COVID-19 vaccine recommendations for children by participant characteristics.

Characteristics	Less than 5 years	5–12 years		
		Extremely likely	Somewhat likely	Not at all likely
All Participants (*n* = 755)	Extremely likely	258	8	2
	Somewhat likely	101	83	7
	Not at all likely	7	36	253
Fully vaccinated (*n* = 578)	Extremely likely	253	8	2
	Somewhat likely	99	70	7
	Not at all likely	7	31	101
Not fully vaccinated (*n* = 175)	Extremely likely	4	0	0
	Somewhat likely	2	12	0
	Not at all likely	0	5	152
Has a child aged less than 5 years (*n* = 99)	Extremely likely	31	0	1
	Somewhat likely	7	7	0
	Not at all likely	0	6	47
Has a child aged 5–12 years (*n* = 183)	Extremely likely	52	0	0
	Somewhat likely	20	16	0
	Not at all likely	5	13	77
Has at least one child of any age (*n* = 557)	Extremely likely	173	5	2
	Somewhat likely	69	60	6
	Not at all likely	7	32	200
Has no children (*n* = 201)	Extremely likely	85	3	0
	Somewhat likely	32	23	1
	Not at all likely	0	4	53
Had a significant COVID-19 infection in the family (*n* = 232)	Extremely likely	81	2	1
	Somewhat likely	25	25	5
	Not at all likely	3	12	78
Had no significant COVID-19 infection in the family (*n* = 519)	Extremely likely	176	6	1
	Somewhat likely	76	57	2
	Not at all likely	4	24	173

The percentage of respondents who were extremely likely to recommend the COVID-19 vaccine to both age groups increased among the fully vaccinated (43.8%), non-parents (42.3%), and those with a significant COVID-19 infection in the family (34.9%). The percentage of respondents who were not at all likely to recommend the vaccine to both age groups increased among the not fully vaccinated (86.8%), parents of a child under 5 years of age (47.5%), 5–12 years of age (42.1%), or a child of any age (35.9%), and among those with a significant COVID-19 infection in the family (33.6%).

### Reasons of concern in recommending the COVID-19 vaccine to children

Table 4 presents the most common concerns from respondents in recommending the COVID-19 vaccine to children under 5 years and 5–12 years. Side effects from the vaccine was the most common concern expressed for both children under 5 years (43.2%) and 5–12 years (35.9%). Other major concerns included rapid vaccine development, not seeing COVID-19 as a threat for children, and not thinking that the vaccine would effectively prevent COVID-19 disease. Among survey participants, 35.4% (*n* = 274) and 41.6% (*n* = 322) did not express any causes of concern in recommending the COVID-19 vaccine to children under 5 years or 5–12 years of age, respectively.

**Table 4:** Top reasons of concern for recommending the COVID-19 vaccine to children^1^.

Reasons of concern	For <5 years	For 5–12 years
Vaccine side effects	335 (43.2%)	278 (35.9%)
Vaccine was developed and approved too quickly	213 (27.5%)	206 (26.6%)
Do not see COVID as a threat to children	159 (20.5%)	164 (21.2%)
Do not think the vaccine will be effective	109 (14.1%)	95 (12.3%)
Concerned that the vaccine can cause infertility	61 (7.9%)	69 (8.9%)
Concerned that the vaccine can alter my child’s DNA	47 (6.1%)	46 (5.9%)
Concerned that government is using vaccine to implant a microchip	22 (2.8%)	26 (3.4%)
Do not believe in vaccines in general	12 (1.6%)	15 (1.9%)
Other	85 (11%)	66 (8.5%)

1 *n* = 274 participants (35.4%) selected “I have no cause of concern” for <5 years old and *n* = 322 participants (41.6%) for 5–12 years old.

## Discussion

Given the expansion of COVID-19 vaccination campaigns in the United States to children between 5 and 11 years in 2021 and then to children under 5 years of age in 2022, it is critically important to understand the motivations and willingness of adults to vaccinate children against COVID-19. This cross-sectional study, performed before approval of the COVID-19 vaccine for children under 5 years of age, is one of the first to examine vaccine willingness for children in this age group in the United States and compare vaccine willingness to children aged 5 to 11 years. The study was performed in a rural county on the United States-Mexico border, which is relevant given the challenges of vaccination coverage in rural areas and the inconsistency of vaccine acceptance based on race and ethnicity (22, 40, 42, 61).

We found that the vaccination status of the respondent was associated with their reported likelihood to recommend the vaccine for children in both age groups; about two-thirds (62%) of fully vaccinated adults were extremely likely to recommend the COVID-19 vaccine to 5–12 year olds, and nearly half (46%) were extremely likely to recommend it to children under 5 years. Furthermore, 44% of fully vaccinated adults reported being extremely likely to recommend the COVID-19 vaccine for both children 5–12 years and under 5 years of age. Conversely, 87% of unvaccinated adults were not likely to recommend the vaccine for children 5–12 years and under 5 years of age.

The strong association between adult vaccination status and vaccine hesitancy is consistent with previous studies that found the vaccination status of parents as the greatest predictor of willingness to vaccinate their children (18, 19, 38, 62–65). Multi-component and dialog-based interventions, targeted to increase knowledge and awareness, were found to be most effective in improving vaccination intentions (66, 67); focusing on vaccination intentions is consistent with the TPB, as intention is the immediate antecedent to the behavior of getting a vaccine (27). These public health communication strategies tailored to emphasize the health risks and public health consequences of not vaccinating against COVID-19 improved vaccination intentions among adults (66, 67). Our findings reaffirm the importance of a multi-sectoral approach to vaccination campaigns and public health messaging with stakeholder collaboration that stresses the importance of vaccine safety and efficacy, debunking myths, illustrating adverse health effects of COVID-19 infection in children to change perceptions of COVID-19 vaccination to improve vaccination intention (68–70).

Latinx adults and adults that speak Spanish as their primary language had higher odds of recommending the vaccine to children in our study. This is consistent with most previous studies showing Latinx parents are less hesitant to vaccinate their children against COVID-19, (38, 40, 44) although not all studies have shown this increased willingness among Latinx populations (19, 65).

This study is one of the first to compare the willingness to vaccinate children among parents with non-parents. Public perceptions and opinions related to the vaccination of children is typically described without taking parental status into account. Thus, we included this aspect in our study to demonstrate whether there is a difference in the hesitancy to vaccinate children based on parental status. Similar to the 50-state COVID-19 survey conducted by the COVID States Project, parents are less likely to recommend the vaccine to children than to non-parents (71). Our study found that 47.5% of parents with a child under 5 years and 42.1% of parents with a child 5–12 years reported being not at all likely to recommend the COVID-19 vaccine to both age groups compared to only 26.4% of adults without children. This difference is likely due to concerns about the vaccine, which parents experience more directly, given that they have children. The major concerns of vaccine recommendation to children reported in our study include vaccine side effects, that the vaccine was developed and approved too quickly, and that COVID-19 is not seen as a threat for children, which are consistent with the most common concerns among parents reported in other studies (62, 72–74).

We found a strong association between the level of concern adults felt about contracting COVID-19 and their reported likelihood of recommending the vaccine to children. Respondents who were moderately or very concerned about getting COVID-19 were more willing to recommend the vaccine to children, and those who were not at all or only a little concerned about getting COVID-19 reported being not likely to recommend the vaccine to children. This association may be informed by the adult’s perceived susceptibility to COVID-19 infection and perceived severity of COVID-19 disease, two constructs from the HBM that are inversely associated with vaccine hesitancy (75). These two HBM constructs may also help explain why adults who had already contracted COVID-19 in the past had lower odds of recommending the vaccine to children. If the adult previously overcame a COVID-19 infection, then they may be less fearful of contracting it again (i.e., have lower perceived susceptibility and perceived severity of COVID-19), and thus would be less likely to recommend the vaccine for their children.

The association between previous infection with COVID-19 and willingness to recommend the vaccine to children did not apply if the infection occurred in another family member. No statistically significant association was found in our study between significant COVID-19 infection of a family member and willingness to recommend the vaccine to children. This is consistent with other studies that found no effect of infection in other household members on willingness to recommend COVID-19 vaccination to children (43, 72). These data suggest that the personal experience of contracting and overcoming COVID-19 was more influential in recommending the vaccine for children than hearing and understanding the experience of others.

Finally, our study found consistency among respondents in their willingness to vaccinate children aged <5 years compared with children aged 5–12 years. Most (78.7%) respondents did not change their willingness to vaccinate children based on age group. This concordance also remained high regardless of vaccination status (73.4% among fully vaccinated, 96.0% among not fully vaccinated) and being a parent (77.7% among parents, 80.1% among non-parents). Given that this study was performed after approval of the vaccine for children 5–12 years old but before approval for children <5 years, this consistency may help predict attitudes and beliefs by adults prior to future vaccine expansions, such as the novel bivalent COVID vaccine.

Limitations to our study include selection bias, limited racial and geographic diversity, and a cross-sectional study design. Given that the survey was distributed by hand, listserv distribution, or personal contacts, there will be underrepresented populations that the survey did not reach. Selection bias is common in all survey studies, including general web surveys with their own self-selection bias (76). The limited geographic and racial makeup of this rural county makes results more difficult to generalize to the broader United States population; however, our findings may be generalizable to U.S-Mexico border communities and the Latinx population in the US. Our study also used cross-sectional data, which does not assess changes in responses, opinions, and attitudes over time. Public opinion and willingness to vaccinate children against COVID-19 likely change throughout the pandemic, depending on the time and setting in which data collection took place.

## Conclusion

Nearly two-thirds, 61.4 and 65.7%, of adults sampled in a rural county in southeast Arizona on the United States-Mexico border were somewhat or extremely likely to recommend COVID-19 vaccination to children under 5 years and 5 to 12 years, respectively. We found a significant increase in the odds of adults being extremely likely to recommend the COVID-19 vaccine to children if they are Latinx, primarily Spanish-speaking, concerned about contracting COVID-19, or are not a parent. Adults were consistent in their willingness to vaccinate children of both age groups, even before the COVID-19 vaccine was approved for children under 5 years of age. Similar to other studies, the greatest concern in vaccinating children against COVID-19 among adults in our study are vaccine side effects, the rapid development of the vaccine, and the perception that COVID-19 is not a threat to children. Given that vaccine hesitancy is one of the major barriers to getting children vaccinated and decreasing nationwide mortality from COVID-19, this study informs public vaccination campaigns that seek to vaccinate children. Public health strategies that target adult vaccinations as an avenue to childhood vaccinations, that include clear communication on the health effects of COVID-19 infection, and that stress COVID-19 vaccine safety and efficacy in children will likely promote vaccine uptake.

## Data availability statement

The raw data supporting the conclusions of this article will be made available by the authors, without undue reservation.

## Ethics statement

Ethical review and approval was not required for the study on human participants in accordance with the local legislation and institutional requirements. The patients/participants provided their written informed consent to participate in this study.

## Author contributions

RD contributed to the survey design, data collection, and manuscript drafting. JH contributed to the survey design, critical revision and edits of the manuscript, and approval of the final version. MM and AB contributed to the data analysis and interpretation. All authors contributed to the article and approved the submitted version.

## Conflict of interest

Authors RD, EH, and JH were employed by Chiricahua Community Health Centers, Inc.

The remaining authors declare that the research was conducted in the absence of any commercial or financial relationships that could be construed as a potential conflict of interest.

## Publisher’s note

All claims expressed in this article are solely those of the authors and do not necessarily represent those of their affiliated organizations, or those of the publisher, the editors and the reviewers. Any product that may be evaluated in this article, or claim that may be made by its manufacturer, is not guaranteed or endorsed by the publisher.
